# Protective factors for psychotic experiences amongst adolescents exposed to multiple forms of victimization

**DOI:** 10.1016/j.jpsychires.2018.06.011

**Published:** 2018-09

**Authors:** Eloise Crush, Louise Arseneault, Terrie E. Moffitt, Andrea Danese, Avshalom Caspi, Sara R. Jaffee, Timothy Matthews, Helen L. Fisher

**Affiliations:** a King's College London, Social, Genetic & Developmental Psychiatry Centre, Institute of Psychiatry, Psychology & Neuroscience, London, UK; b Department of Psychology and Neuroscience, Duke University, Durham, NC, USA; c Department of Psychiatry and Behavioral Sciences, Duke University, Durham, NC, USA; d King's College London, Department of Child & Adolescent Psychiatry, Institute of Psychiatry, Psychology & Neuroscience, London, UK; e National & Specialist CAMHS Trauma and Anxiety Clinic, South London and Maudsley NHS Foundation Trust, London, UK; f Department of Psychology, University of Pennsylvania, Philadelphia, PA, USA

**Keywords:** Adolescence, Exercise, Poly-victimization, Psychosis, Resilience, Social support

## Abstract

Experiencing multiple types of victimization (poly-victimization) during adolescence is associated with the onset of psychotic experiences (such as hearing voices, having visions, or being extremely paranoid). However, many poly-victimized adolescents will not develop such subclinical phenomena and the factors that protect them are unknown. This study investigated whether individual, family, or community-level characteristics were associated with an absence of psychotic experiences amongst poly-victimized adolescents. Participants were from the Environmental Risk (E-Risk) Longitudinal Twin Study, a nationally-representative cohort of 2232 UK-born twins. Exposure to seven different types of victimization between ages 12–18 was ascertained using a modified version of the Juvenile Victimization Questionnaire at age 18. Adolescents were also interviewed about psychotic experiences at age 18. Protective factors were measured at ages 12 and 18. We found that exposure to poly-victimization during adolescence was associated with age-18 psychotic experiences (OR = 4.62, 95% CI 3.59–5.94, P < 0.001), but more than a third of the poly-victimized adolescents reported having no psychotic experiences (40.1%). Greater social support was found to be protective against adolescent psychotic experiences even amongst those exposed to poly-victimization. Engaging in physical activity and greater neighborhood social cohesion were also associated with a reduced likelihood of age-18 psychotic experiences in the whole sample, with non-significant trends in the poly-victimized group. Increasing social support and promoting physical activity appear to be important areas for future research into the development of preventive interventions targeting adolescent psychotic experiences. This adds further weight to calls to increase the promotion of these factors on a public health scale.

## 1 Background

Psychotic experiences (such as hearing voices, having visions, and feeling extremely paranoid) occurring during late adolescence have been found to precede the development of psychotic disorders (Dominguez et al., 2011) and a wide range of other severe mental health problems including suicide attempts (McGrath et al., 2016). Psychotic experiences during this developmental stage have also been shown to be associated with greater psychiatric comorbidity than psychotic phenomena occurring during late childhood (Kelleher et al., 2012a). We must, therefore, develop a better understanding of how to prevent the development of psychotic experiences in adolescence.

Exposure to victimization (e.g., physical abuse, sexual abuse, bullying by peers) during adolescence has been found to be a major risk factor for the onset of psychotic experiences in this period (Kelleher et al., 2013). Moreover, experiencing two or more different types of victimization (often referred to as poly-victimization; Finkelhor et al., 2007) has been associated with the highest risk of psychotic phenomena emerging (Arseneault et al., 2011). Identifying multi-level factors that are protective against the development of psychotic experiences, particularly in this high-risk group of poly-victimized adolescents, may be especially relevant for prevention efforts.

There has been little research to date on protective factors for psychotic phenomena, with the vast majority of studies focusing on factors that increase rather than decrease risk. This is despite calls for a shift towards investigating what enables some victimized individuals to avoid developing psychotic experiences in the hope that such findings could inform preventive interventions (Morgan and Gayer-Anderson, 2016). In a recent study (Crush et al., 2017), we found that having a relatively high IQ, a more positive atmosphere at home, and higher levels of neighborhood social cohesion (meaning neighbors get along well and share common values) were associated with a reduced likelihood of psychotic symptoms emerging at age 12 amongst poly-victimized children. The current paper extends these findings by considering whether similar factors are protective amongst individuals exposed to poly-victimization during adolescence in relation to psychotic experiences at age 18. Moreover, the wider literature suggests that some additional factors may be protective during this period including: positive coping strategies (Jalbrzikowski et al., 2014; Kommescher et al., 2016), engagement in physical activity (Callaghan, 2004; Suetani et al., 2017), and social support in terms of both perceived practical and emotional support from others and the number of social connections (Gayer-Anderson et al., 2015; Gayer-Anderson and Morgan, 2013; Hodges et al., 1999), and therefore these putative protective factors will also be investigated in the current study.

This paper utilises data from a large, nationally-representative UK birth cohort to explore whether individual (higher IQ, positive coping strategies, higher levels of physical activity), family (positive home atmosphere), community (socially cohesive neighborhood), and cross-level (greater perceived social support) factors are associated with a reduced likelihood of developing psychotic experiences in our population sample. We considered whether any of these factors were found to be protective in the context of poly-victimization during adolescence by (i) repeating analyses in this sub-sample, and (ii) testing for interactions between poly-victimization and putative protective factors in relation to an absence of age-18 psychotic experiences in the whole sample.

## 2 Materials and methods

### 2.1 Study cohort

Participants were members of the Environmental Risk (E-Risk) Longitudinal Twin Study, which tracks the development of a nationally-representative birth cohort of 2232 British twin children born in England and Wales in 1994–1995. Full details about the sample are reported elsewhere (Moffitt and The E-Risk Team, 2002), and in the Supplementary Materials. Briefly, the E-Risk sample was constructed in 1999–2000, when 1116 families with same-sex 5-year-old twins (93% of those eligible) participated in home-visit assessments. Families were recruited to represent the UK population of families with newborns in the 1990s, based on residential location throughout England and Wales and mothers' age. E-Risk families are representative of UK households across the spectrum of neighborhood-level deprivation (see Supplementary Materials). The sample comprised 56% monozygotic and 44% dizygotic twin pairs, and sex was evenly distributed within zygosity (49% male). Follow-up home-visits were conducted when children were aged 7, 10, 12, and 18 years (participation rates were 98%, 96%, 96%, and 93% respectively). The Joint South London and Maudsley and the Institute of Psychiatry Research Ethics Committee approved each phase of the study. Parents gave informed consent and twins gave assent between 5 and 12 years and then informed consent at age 18.

### 2.2 Measures

#### 2.2.1 Individual-level protective factors

##### 2.2.1.1 IQ

The Wechsler Intelligence Scale for Children (WISC) (Wechsler, 2003) was used to assess IQ at age 12. Children were administered 3 tasks: matrix reasoning, information and digit span. The three scores were combined to create an overall scale and then standardized with a mean of 100 and standard deviation of 15.

##### 2.2.1.2 Coping strategies

Coping was assessed at age 18 by asking participants about which strategies they used when experiencing stress in relation to finances, relationships, college or work. Four positively-coded items (“talk with other people about it”, “talk with a therapist or counsellor”, “exercise” and “take steps to solve the problem”) were combined to create a scale with higher scores reflecting more positive coping strategies.

##### 2.2.1.3 Physical activity

At age 18, participants completed the Stanford Brief Activity Survey (SBAS; Stanford University, 2001). The SBAS contains 2 items, the first item relates to the extent of physical activity engaged in at work, school or college and the second refers to physical activity during leisure time. Both questions were rated on a 5-point scale: inactive, low intensity, moderate intensity, hard intensity and very hard intensity. The scales were then combined to derive an overall activity measure (Taylor-Piliae et al., 2010). For the current study, we used a binary variable for the analysis which compared those who were inactive (rating of 1) to those who were active (rating of 2–5).

#### 2.2.2 Family-level protective factors

##### 2.2.2.1 Atmosphere at home

The creation of the atmosphere at home measure has been previously documented (Kim-Cohen et al., 2006). Briefly, it was derived from the Coder's Impression Inventory, which is based on the Home Observation for Measurement of the Environment (HOME) (Bradley and Caldwell, 1977) and the University of Washington Parenting Clinic Questionnaire (Parent–Child Observations) (Webster-Stratton, 1998). The Coder's Impression Inventory was rated by interviewers, who had undergone four-day training, immediately following the study visit with mothers when the twins were aged 12. This measure comprised items representing the state of the home (e.g., ‘Are visible rooms of the house clean?’), stimulation (e.g., ‘Is the children's art displayed in the home?’), happiness (e.g., ‘Is this a happy home?’), and chaos (e.g., ‘Is the house chaotic or overly noisy?’). The internal consistency between items was α = 0.76.

#### 2.2.3 Community-level protective factors

##### 2.2.3.1 Social cohesion

Social cohesion was estimated via a postal survey sent to residents living alongside E-Risk families when participants were aged 13–14 (Odgers et al., 2009, 2012). Survey respondents, who were typically living on the same street or within the same apartment block as the participants in our study, reported on various characteristics of their immediate neighborhood. Five items (each coded 0–4) were assessed including the questions: “is this a close-knit neighborhood”, “do you think people in this neighborhood can be trusted”, “do you share the same values”, etc. We derived a total scale by summing the answers to all 5 questions with higher scores indicative of greater social cohesion.

#### 2.2.4 Cross-level protective factors

##### 2.2.4.1 Social support

Social support was assessed using the Multidimensional Scale of Perceived Social Support (MSPSS), which assesses individuals' access to supportive relationships with family, friends and significant others (Zimet et al., 1988). The 12 items in the MSPSS consist of statements such as ‘‘There is a special person who is around when I am in need’’ and ‘‘I can count on my friends when things go wrong’’. Participants rated these statements as ‘‘not true’’ (0), ‘‘somewhat true’’ (1) or ‘‘very true’’ (2). We summed scores to produce an overall social support scale with higher scores reflecting greater social support (internal consistency: α = 0.88). In addition, each of the three sub-scales was utilized separately to examine whether social support from either family, friends or significant others was found to be specifically protective.

#### 2.2.5 Adolescent psychotic phenomena

The present study uses two measures of psychotic phenomena which were both obtained from private interviews when participants were aged 18. Our primary outcome was a self-report measure of adolescent psychotic *experiences* which reflects the methodology used by many groups in the psychosis prodromal research field (Loewy et al., 2011). At age 18, each E-Risk participant was privately interviewed by a research worker about 13 psychotic experiences occurring since age 12. Seven items pertained to delusions and hallucinations and this interview has been described in detail previously (Polanczyk et al., 2010) and in the Supplementary Materials. Six items pertained to unusual experiences which drew on item pools since formalized in prodromal psychosis instruments including the PRIME-screen and SIPS (Loewy et al., 2011). These included “I worry that my food may be poisoned” and “My thinking is unusual or frightening”. Interviewers coded each item 0, 1, 2 indicating respectively “not present”, “probably present”, and “definitely present”. All 13 items were summed to create a psychotic experiences scale (range = 0–18, M = 1.19, SD = 2.58). Just over 30% of participants had at least one psychotic experience between ages 12 and 18 (n = 623, 30.2%). This is similar to the prevalence of self-reported psychotic experiences in other community samples of teenagers and young adults (Kelleher et al., 2012b; Yoshizumi et al., 2004). The presence (30.2%) versus absence (69.8%) of one or more “definitely present” psychotic experiences is used as a dichotomous dependent variable in the current study.

We additionally examined clinically-verified adolescent psychotic *symptoms* as a secondary outcome, using the same methodology as used at age 12 in this cohort (Polanczyk et al., 2010). Responses to the seven hallucination/delusion items were verified by a team of clinicians, including child and adolescent psychiatrists, to capture more clinically pertinent psychotic symptoms (see Supplementary Materials). At age 18, 2.9% (N = 59) of participants were designated as having experienced at least 1 definite psychotic *symptom.*

#### 2.2.6 Adolescent poly-victimization

At age 18, participants were interviewed about exposure to a range of adverse experiences between 12 and 18 years using the Juvenile Victimization Questionnaire, 2nd revision (JVQ-R2) (Finkelhor et al., 2011) adapted as a clinical interview, which has been outlined in a previous paper (Fisher et al., 2015) and described more fully in the Supplementary Materials. Each twin was interviewed by a different research worker, and each JVQ question was asked for the period ‘since you were 12’. Age 12 is a salient age for our participants because it is the age when British children leave primary school to enter secondary school. Our adapted JVQ comprised 45 questions covering 7 different forms of victimization: maltreatment, neglect, sexual victimization, family violence, peer/sibling victimization, internet/mobile phone victimization, and crime victimization. The worst experience (according to the participant) for each victimization type was rated by trained coders using a 6-point scale: 0 = not exposed, then 1–5 for increasing levels of severity. The adolescent poly-victimization variable was derived by summing all victimization experiences that received a code of ‘4’ or ‘5’ (i.e., severe exposure): 64.6% of adolescents had zero severe victimization experiences; 19.2% had 1; 9.4% had 2; 4.5% had 3; 1.5% had 4; 0.5% had 5; and 0.2% had 6 severe victimization experiences. Due to small numbers in some of the groups, we collapsed this variable into ‘0’ not victimized, ‘1’ experienced 1 type of severe victimization, and ‘2’ poly-victimized (experienced 2 or more types of severe victimization).

#### 2.2.7 Potential confounders

Family socioeconomic status (SES) was measured via a composite of parental income (total household), education (highest for mother/father), and occupation (highest for mother/father) when children were aged 5 (Trzesniewski et al., 2006), and was categorized into tertiles (i.e., low-, medium-, and high-SES). Mothers reported on family history of DSM disorders (Weissman, 2000) in private interviews when participants were aged 12, which was converted to a proportion (0–1.0) of family members with a history of psychiatric disorder (Milne et al., 2008). Childhood psychotic symptoms pertaining to seven delusions and hallucinations were measured when children were aged 12 during private interviews. Items and interviewer notes were assessed by a psychiatrist expert in schizophrenia, a psychologist expert in interviewing children, and a child and adolescent psychiatrist to verify the validity of the symptoms (Polanczyk et al., 2010). A total of 5.9% of children reported experiencing at least one definite psychotic symptom at age 12 (N = 125). A variable was also created for the presence vs. absence of any childhood mental health problems to capture children who met criteria for extreme anxiety, clinically-relevant depression symptoms, attention deficit hyperactivity disorder (ADHD), or conduct disorder by age 12 (see Supplementary Materials).

### 2.3 Statistical analysis

Analyses were conducted in STATA 11.2 (Stata-Corp, College Station, TX). Because each study family contains two children, all statistical analyses were corrected conservatively for the non-independence of twin observations by using tests based on the Huber/White variance estimator (Williams, 2000). We used logistic regression to test the associations between individual, family, community, and cross-level factors and absence of age-18 psychotic experiences in (i) the whole sample and (ii) the sub-sample with adolescent poly-victimization. We also tested for interactions between poly-victimization and any factors found to be associated with an absence of age-18 psychotic experiences in the poly-victimized group using logistic regression to examine whether these factors were specifically protective in relation to poly-victimization exposure. All of these analyses were subsequently adjusted for gender, family SES, family psychiatric history, age-12 psychotic symptoms, and childhood mental health problems. Sensitivity analyses were also conducted using the rarer clinically-verified psychotic symptoms at age 18 as the outcome variable for analyses conducted in the whole sample.

## 3 Results

### 3.1 Are any individual, family or community-level factors associated with the absence of age-18 psychotic experiences in the whole sample?

First, we considered whether any of the factors were associated with a reduced likelihood of psychotic experiences emerging at age 18 in the whole sample (Table 1). We found that engaging in physical activity, higher levels of social cohesion, and greater levels of social support were all associated with a reduced likelihood of psychotic experiences being reported at age 18 when controlling for potential confounders. Furthermore, multivariate models including the above significant predictors showed that independent associations were found for engaging in physical activity (OR = 0.59, 95% CI 0.36–0.96, *P* = 0.035), increased social support (OR = 0.91, 95% CI 0.89–0.94, *P* < 0.001), and higher levels of social cohesion (OR = 0.77, 95% CI 0.60–0.98, *P* = 0.035). When considered individually, each social support type was found to be protective: family (OR = 0.80, 95% CI 0.76–0.86, *P* < 0.001), friends (OR = 0.83, 95% CI 0.78–0.88, *P* < 0.001), and significant others (OR = 0.92, 95% CI 0.87–0.97, *P* = 0.004), after controlling for all other significant factors. Broadly similar results were found when repeating analyses using clinically-verified psychotic symptoms (Table 2).

**Table 1:** Associations between individual, family, and community factors in adolescence and age-18 psychotic experiences in the full sample.

Protective Factors	Whole Sample (N = 2063)			
	No Psychotic Experiences N = 1440 M (SD)	Psychotic Experiences N = 623 M (SD)	Unadjusted OR (95% CI)	Adjusted OR^a^ (95% CI)
IQ at age 12	101.4 (14.9)	97.5 (14.6)	**0.98 (0.98–0.99)**	0.99 (0.99–1.00)
Physically active at age 18, n (%)	1396 (96.9)	575 (92.7)	**0.40 (0.26–0.62)**	**0.49 (0.30–0.77)**
Positive coping strategies at age 18	3.0 (1.7)	3.0 (1.7)	0.98 (0.92–1.04)	1.02 (0.95–1.08)
Atmosphere at home at age 12	24.2 (5.4)	22.9 (5.6)	**0.96 (0.94–0.98)**	0.99 (0.97–1.02)
Social cohesion at age 13/14	2.3 (0.5)	2.2 (0.5)	**0.66 (0.53–0.82)**	**0.78 (0.61–0.99)**
Social support at age 18	21.3 (3.9)	19.3 (5.0)	**0.90 (0.88–0.92)**	**0.91 (0.89–0.94)**

a Adjusted for family socioeconomic status, family psychiatric history, child's gender, age-12 psychotic symptoms, and other mental health problems at age 12. All analyses account for the non-independence of twin observations. CI, confidence interval. IQ, intelligence quotient. M, mean. OR, odds ratio. SD, standard deviation. Bold text indicates p < 0.05.

**Table 2:** Associations between individual, family, and community factors in adolescence and age-18 clinically-verified psychotic symptoms in the full sample.

Protective Factors	Whole Sample (N = 2063)		
	No Psychotic Symptoms N = 2004 M (SD)	Psychotic Symptoms N = 59 M (SD)	Unadjusted OR (95% CI)
IQ at age 12	100.3 (14.9)	97.6 (15.7)	0.99 (0.97–1.01)
Physically active at age 18, n (%)	1917 (95.9)	53 (89.8)	**0.38 (0.16–0.92)**
Coping strategies at age 18	3.0 (1.7)	3.4 (1.8)	1.15 (0.99–1.35)
Atmosphere at home at age 12	23.9 (5.5)	22.0 (5.5)	**0.95 (0.92–0.99)**
Social cohesion at age 13/14	2.2 (0.5)	2.1 (0.6)	**0.58 (0.34–0.99)**
Social support at age 18	20.8 (4.3)	18.2 (6.1)	**0.91 (0.86–0.95)**

All analyses account for the non-independence of twin observations. Due to the small number of individuals with psychotic symptoms, all analyses are presented without adjustment for potential confounders. CI, confidence interval. IQ, intelligence quotient. M, mean. OR, odds ratio. SD, standard deviation. Bold text indicates p < 0.05.

### 3.2 Is poly-victimization during adolescence associated with age-18 psychotic experiences?

Psychotic experiences at age 18 were more commonly reported by adolescents who were exposed to one type of victimization (41.0% vs. 26.2%; OR = 1.96, 95% CI 1.57–2.45, *P* < 0.001), and even more so amongst those exposed to two or more types (59.9% vs. 24.4%; OR = 4.62, 95% CI 3.59–5.94, *P* < 0.001) compared to those not exposed to any victimization between 12 and 18 years. Given that the poly-victimized group had the greatest likelihood of reporting age-18 psychotic experiences we focussed our analysis on these high-risk adolescents. This association with poly-victimization remained after controlling for family SES (OR = 4.36, 95% CI 3.38–5.62, *P* < 0.001), family psychiatric history (OR = 4.33, 95% CI 3.34–5.61, *P* < 0.001), age-12 psychotic symptoms (OR = 4.31, 95% CI 3.33–5.60, *P* < 0.001), and other mental health problems at age 12 (OR = 4.12, 95% CI 3.18–5.35, *P* < 0.001). It also did not significantly differ for boys and girls (sex interaction: OR = 1.73, 95% CI 0.75–3.99, *P* = 0.197), and therefore we present all further results for both sexes together. In total, over a third of poly-victimized adolescents reported not having any psychotic experiences at age 18 (40.1%).

### 3.3 Are individual, family, and community-level factors associated with the absence of age-18 psychotic experiences amongst poly-victimized adolescents?

Next, we explored whether the factors significantly associated with an absence of psychotic experiences in the whole sample were protective amongst adolescents exposed to multiple forms of victimization (Table 3). Only greater social support at age 18 was found to be significantly associated with a reduced likelihood of age-18 psychotic experiences amongst poly-victimized adolescents (OR = 0.93, 95% CI 0.88–0.98, *P* = 0.011) after adjustment for a range of confounders. Physical activity also showed a strong trend with a reduced likelihood of psychotic experiences in the poly-victimized group after controlling for all confounders, albeit this association failed to meet conventional levels of statistical significance (OR = 0.49, 95% CI 0.18–1.26, *P* = 0.134).

**Table 3:** Associations between potential protective factors and age-18 psychotic experiences amongst adolescents exposed to poly-victimization.

Protective Factors	Poly-victimized adolescents (N = 334)			
	No Psychotic Experiences N = 134 M (SD)	Psychotic Experiences N = 200 M (SD)	Unadjusted OR (95% CI)	Adjusted OR^a^ (95% CI)
Physically active at age 18, n (%)	128 (95.5)	178 (89.5)	0.40 (0.15–1.03)	0.48 (0.18–1.26)
Social cohesion at age 13/14	2.2 (0.4)	2.1 (0.6)	0.71 (0.45–1.13)	0.86 (0.52–1.42)
Social support at age 18	20.1 (5.0)	18.1 (5.4)	**0.92 (0.88–0.97)**	**0.93 (0.88–0.98)**

a Adjusted for family socioeconomic status, family psychiatric history, child's gender, age-12 psychotic symptoms, and other mental health problems at age 12. All analyses account for the non-independence of twin observations. CI, confidence interval. IQ, intelligence quotient. M, mean. OR, odds ratio. SD, standard deviation. Bold text indicates p < 0.05.

When considering the social support sub-scales separately, two of them were significantly associated with an absence of psychotic experiences among poly-victimized adolescents: support from family (OR = 0.83, 95% CI 0.73–0.94, *P* = 0.002) and friends (OR = 0.89, 95% CI 0.81–0.98, *P* = 0.021). Finally, we tested for an interaction between social support and poly-victimization to ascertain whether this was particularly protective against adolescent psychotic experiences in the context of poly-victimization exposure. However, we did not find this interaction to be significant (interaction OR = 1.00, 95% CI 0.94–1.07, *P* = 0.816).

## 4 Discussion

This is the first study to investigate putative protective factors in relation to psychotic experiences amongst adolescents. We found that engaging in physical activity, greater social support, and more social cohesion within the surrounding neighborhood were associated with an absence of psychotic experiences at age 18 in this general population sample; these associations remained after controlling for a range of confounders including earlier mental health problems at age 12. These factors, together with a positive atmosphere at home, were also found to be associated with an absence of the rarer clinically-verified psychotic symptoms in the whole sample. However, when considering factors that were protective amongst the high-risk group exposed to poly-victimization, we only found greater social support to be protective against adolescent psychotic experiences.

The most notable finding is that social support consistently comes through as being independently associated with a reduced likelihood of adolescent psychotic experiences even in the context of poly-victimization, as well as in relation to the clinically-verified age-18 psychotic symptoms in the whole sample. The social support measure in this study is based upon adolescents' perceptions of the social support they receive from friends, family and significant others, and thus captures both subjective views of availability and functional aspects of social support (Valtorta et al., 2016). Our findings are consistent with previous research which has found social support to be associated with positive emotional and behavioural adjustment during adolescence, perhaps due to improvements in self-esteem (Smith et al., 2006; Turner et al., 2015) or reducing loneliness (Lim et al., 2018; Sündermann et al., 2014). Self-esteem is particularly relevant given that low self-esteem has been found to be predictive of psychotic phenomena in non-clinical populations previously (Krabbendam et al., 2002) and to mediate the association between victimization and psychotic experiences during adolescence (Fisher et al., 2013).

It has also been proposed that social support may play an important role in buffering stress levels (Cohen and Wills, 1985; Stadler et al., 2010) and relatedly has been found to be a key coping strategy for adolescents (Eschenbeck et al., 2007), which may also explain why social support was protective for those adolescents exposed to multiple forms of victimization. In addition, our findings are consistent with a study that found that social support may buffer the effects of some forms of victimization on adult psychosis (Gayer-Anderson et al., 2015). These findings suggest that social support is an important area to focus on to prevent the emergence of psychotic experiences in adolescence, which requires further research and clinical attention. However, it is also possible that adolescents who demonstrate resilience in the face of adversity are more attractive to others and thus have more friends and elicit greater social support so further investigation of the direction in which this association is operating is required.

Being physically active during work and leisure time was found to be independently associated with lower rates of adolescent psychotic experiences in the whole sample and also showed a strong (albeit non-significant) protective trend in the poly-victimized group. Our findings are consistent with a number of recent studies which have highlighted that inactivity during adolescence is associated with psychotic phenomena in early adulthood (Suetani et al., 2017) and the benefit of exercise interventions for reducing psychotic phenomena amongst those at risk for psychosis as well as clinical groups suffering from psychotic disorders and also depression (Dauwan et al., 2016; Firth et al., 2015, 2016). In terms of mechanisms through which exercise may reduce the likelihood of psychotic phenomena, it has been suggested these could be biological (stress buffering), psycho-social (social connectedness) and psychological (self-esteem), albeit further research is needed in relation to physical activity and psychotic phenomena to understand the association and mechanisms in more detail (Knowles, 2017). Finally, it is important to note that as our finding on physical activity and psychotic experiences is based on cross-sectional analyses we cannot draw any conclusions regarding the direction of the association. It is also plausible that negative symptoms (such as anhedonia and avolition), which may precede or accompany the positive psychotic experiences that we measured, could explain the lack of engagement in physical activity amongst those reporting psychotic phenomena.

### 4.1 Limitations

Some limitations warrant consideration. Firstly, despite this being a large cohort, the number of poly-victimized adolescents was reasonably modest (N = 334) and this may have limited our ability to detect some associations between the proposed protective factors and a reduced likelihood of developing psychotic experiences, and particularly interaction effects. These analyses thus warrant replication in even larger cohorts of victimized adolescents. Additionally, the self-report measure of adolescent psychotic experiences utilized for most of the analyses may have captured genuine experiences (e.g., being followed by a stranger) as well as psychotic phenomena (e.g., being followed by an angel). This may have led to inflated associations for adolescent psychotic experiences, though it is reassuring that the effect sizes were fairly similar to those produced for clinically-verified psychotic symptoms. Relatedly, the low numbers of individuals with clinically-verified psychotic symptoms meant that we lacked power to detect significant associations when using this outcome and were unable to look at it in the poly-victimized group. It is also important to note that it was not possible to identify the specific timing of victimization exposure within the 6-year period and therefore we were not able to look at timing in further detail.

The social support scale used is a self-report measure reflecting individuals' perceptions of support from friends, family and significant others, thus it is possible that individuals who develop psychotic experiences may perceive their support levels to be lower than the support that is actually available and therefore we welcome replication of our findings amongst cohorts with co-informant measures of social support in order to understand this association more clearly. Finally, the E-Risk cohort comprises twins, and whether findings from twin studies generalize to singletons is sometimes contested. However, the adolescents in our study reported a similar prevalence of psychotic experiences (Horwood et al., 2008; Scott et al., 2006; Yoshizumi et al., 2004) and victimization (Radford et al., 2013) to those found for singletons, and are representative of UK families in terms of geographic and socioeconomic distribution (Moffitt and The E-Risk Team, 2002; CACI, 2006).

## 5 Conclusion

Greater social support, higher levels of neighborhood social cohesion, and engaging in physical activity were all found to be associated with a reduced likelihood of having adolescent psychotic experiences in the full sample. Greater social support (and to a non-significant degree physical activity) also showed strong protective effects in the context of poly-victimization. Our findings have implications for the potential focus and timing of early interventions. Our research suggests interventions focused on improving individual's social support from friends and family or how they perceive existing social support around them as well as increasing physical activity could be effective in reducing psychotic phenomena, and that these interventions should be targeted at poly-victimized adolescents who are at greatest risk for developing psychotic experiences. It is encouraging that increasing the availability of social support and improving physical activity levels constitute interventions that would be feasible to implement on both the population-level and amongst high-risk groups.

## Financial support

The E-Risk Study is funded by the UK Medical Research Council (G1002190). Additional support was provided by the National Institute of Child Health and Human Development (HD077482); the Jacobs Foundation; the Avielle Foundation; a research grant from the National Society for Prevention of Cruelty to Children (NSPCC) and Economic and Social Research Council; a Medical Research Council Studentship to EC; and an MQ Transforming Mental Health (MQ14F40) Fellows Award to HLF. LA is the Mental Health Leadership Fellow for the UK ESRC.

## Conflicts of interest

None.

## Appendix A Supplementary data

Crush et al Supplementary Materials JPsychRes REVISED Crush et al Supplementary Materials JPsychRes REVISED
