# Supplementary material for: Protective factors for psychotic experiences amongst adolescents exposed to multiple forms of victimization
Source: J Psychiatr Res. 2018 Sep;104:32–8. doi: 10.1016/j.jpsychires.2018.06.011 (PMC6109202; doi:10.1016/j.jpsychires.2018.06.011)
Supplement: Crush et al Supplementary Materials JPsychRes REVISED [file mmc1.doc]

**Supplementary Materials**

**Study Cohort**

Participants were members of the Environmental Risk (E-Risk) Longitudinal Twin Study, which tracks the development of a nationally-representative birth cohort of 2232 British twin children. The sample was drawn from a larger cohort of twins born in England and Wales in 1994-1995.^1^ Full details about the sample are reported elsewhere.^2^ Briefly, the E-Risk sample was constructed in 1999-2000, when 1116 families with same-sex 5-year-old twins (93% of those eligible) participated in home-visit assessments. Families were recruited to represent the UK population of families with newborns in the 1990s, based on residential location throughout England and Wales and mothers’ age. Teenaged mothers with twins were over-selected to replace high-risk families who were selectively lost to the register through non-response. Older mothers having twins via assisted reproduction were under-selected to avoid an excess of well-educated older mothers. E-Risk families are representative of UK households across the spectrum of neighborhood-level deprivation: 25.6% of E-Risk families live in “wealthy achiever” neighborhoods compared to 25.3% of households nation-wide; 5.3% vs 11.6% live in “urban prosperity” neighborhoods; 29.6% vs 26.9% live in “comfortably off” neighborhoods; 13.4% vs 13.9% live in “moderate means” neighborhoods; and 26.1% vs 20.7% live in “hard-pressed” neighborhoods.^3,4^ E-Risk families under-represent “urban prosperity” neighborhoods because such households are likely to be childless. The sample comprised 56% monozygotic and 44% dizygotic twin pairs, and sex was evenly distributed within zygosity (49% male). All families were English speaking, and the majority (93.7%) were White.

Follow-up home visits were conducted when children were 7 years (98% of the 1116 E-Risk Study families participated), 10 years (96% participation), 12 years (96% participation) and 18 years (93% participation). Home visits at ages 5, 7, 10, and 12 years included assessments with participants as well as their mother (or primary caretaker); the home visit at age 18 included interviews only with the participants. Each twin participant was assessed by a different interviewer. The average age of the twins at the time of the age 18 assessment was 18.4 years (*SD*=0.36); all interviews were conducted after the 18^th^ birthday. There were no differences between those who did and did not take part at age 18 in terms of socioeconomic status (SES) assessed when the cohort was initially defined (χ^2^=0.86, *p*=0.65), age-5 IQ scores (*t*=0.98, *p*=0.33), or age-5 internalizing or externalizing behavior problems (*t*=0.40, *p*=0.69 and *t*=0.41, *p*=0.68, respectively). The Joint South London and Maudsley and the Institute of Psychiatry Research Ethics Committee approved each phase of the study. Parents gave informed consent, and participants gave assent at ages 5-12 and informed consent at age 18.

**Measure of clinically-verified psychotic symptoms**

E-Risk Study members were visited by mental health trainees or professionals when they were aged 18.[^5^](#_ENREF_5) Each adolescent was privately interviewed about whether they had experienced 7 psychotic symptoms pertaining to delusions and hallucinations since age 12, with items including “have other people read your thoughts?,” “have you thought you were being followed or spied on?,” and “have you heard voices that other people cannot hear?.” This interview has been described in detail previously,^5^ and was used at age 12 to estimate childhood psychotic symptoms. The item choice was guided by the Dunedin Study’s age-11 interview protocol[^6^](#_ENREF_6) and an instrument prepared for the Avon Longitudinal Study of Parents and Children.[^7^](#_ENREF_7) At age 18 (and 12) interviewers coded each experience 0, 1, 2 indicating respectively “not a symptom,” “probable symptom,” and “definite symptom.” A conservative approach was taken in designating an adolescent’s report as a symptom. First, the interviewer probed using standard prompts designed to discriminate between experiences that were plausible (e.g., “I was followed by a man after school”) and potential symptoms (e.g., “I was followed by an angel who guards my spirit”), and wrote down the adolescent’s narrative description of the experience. Second, items and interviewer notes were assessed by psychologists’ expert in assessing psychosis, and a child and adolescent psychiatrist to verify the validity of the symptoms. Third, because Study members were twins, experiences limited to the twin relationship (e.g., “My twin and I often know what each other are thinking”) were coded as “not a symptom”. Adolescents were only designated as having psychotic symptoms if they reported at least one definite and verified symptom. At age 18, 2.9% (N=59) of adolescents reported having psychotic symptoms since age 12. This is somewhat lower than the prevalence of psychotic symptoms in this sample at age 12 (5.9%, N=125), consistent with the attenuation of psychotic symptoms documented from childhood to adulthood. Furthermore, our psychotic symptom measure has good construct validity, sharing many of the genetic, social, neurodevelopmental, and behavioural risks factors and correlates as adult schizophrenia.[^5^](#_ENREF_5)

**Measure of childhood mental health problems**

A variable for childhood mental health problems was derived to capture children who met criteria for extreme anxiety, clinically-relevant depression symptoms, attention deficit hyperactivity disorder (ADHD), or conduct disorder by age 12. Anxiety was assessed when children were aged 12, via private interviews using the 10-item version of the Multidimensional Anxiety Scale for Children (MASC).^10^ An extreme anxiety group was formed with children who scored at or above the 95th percentile (N = 129, 6.1%). Depression symptoms were assessed at age 12 using the Children’s Depression Inventory (CDI).^11^ Children who scored 20 or more were deemed to have clinically significant depressive symptoms (N = 74, 3.5%). ADHD was assessed using the DSM-IV and the requirement of symptom onset prior to age 12 was met if parents or teachers reported more than 2 ADHD symptoms at ages 5, 7, 10, or 12 years. We derived diagnoses of conduct disorder on the basis of mothers’ and teachers’ reports of children’s behaviour problems using the Achenbach family of instruments and additional DSM-IV items assessing conduct disorder which have previously been described.^12^ Conduct disorder was assumed present if it was diagnosed at ages 5, 7, 10 or 12 years. This variable was dichotomised to distinguish between the presence of any of the above mental health problems (coded 1) versus the absence of any age 12 mental health problems (coded as 0).

**Assessment of victimization in adolescence**

We have previously reported evidence on the reliability and validity of our measurement of adolescent victimization.^13^ Here we summarize the method. Participants were interviewed about experiences between 12-18 years using the Juvenile Victimization Questionnaire (JVQ),^14,15^ adapted as a clinical interview. The JVQ has good psychometric properties^16^ and was used in the U.K. National Society for the Prevention of Cruelty to Children national survey,^17,18^ thereby providing benchmark values for comparisons with our cohort.

Within each pair of twins in our cohort, co-twins were interviewed separately by a different research worker and were assured of the confidentiality of their responses. The participants were advised that confidentiality would only be broken if they told the research worker that they were in immediate danger of being hurt, and in such situations the project leader would be informed and would contact the participant to discuss a plan for safety. We assessed 7 different forms of victimization: maltreatment, neglect, sexual victimization, family violence, peer/sibling victimization, internet/mobile phone victimization, and crime victimization. Each JVQ question was asked for the period ‘since you were 12’. Participants were given the option to say “yes” or “no” as to whether each type of victimization had occurred in the reporting period. Research workers could rate each item “maybe” if the participant seemed unsure or hesitant in their response or they were not convinced that the participant understood the question or was paying attention. Items rated as “maybe” were recoded as “no” or “yes” by the rating team based on the notes provided by the research workers. When insufficient notes were available, these responses were recoded conservatively as a “no”. Consistent with the JVQ manual,^14,15^ participants were coded as 1 if they reported any experience within each type of victimization category, or 0 if none of the experiences within the category were endorsed. If an experience was endorsed within a victimization category, follow-up questions were asked concerning how old the participant was when it (first) happened, whether the participant was physically injured in the event, whether the participant was upset or distressed by the event; and how long it went on for (by marking the number of years on a Life History Calendar.^19^ In addition, the interviewer wrote detailed notes based on the participant’s description of the worst event. If multiple experiences were endorsed within a victimization category, the participant was asked to identify and report about their worst experience.

All information from the JVQ interview was compiled into victimization dossiers. Using these dossiers, each of the seven victimization categories was rated by an expert in victimology and 3 other members of the E-Risk team who were trained on using the rating criteria. Ratings were made using a 6-point scale: 0 = not exposed, then 1-5 for increasing levels of severity. The anchor points for these ratings were adapted from the coding system used for the Childhood Experience of Care and Abuse interview (CECA),^20,21^ which has good inter-rater reliability.^20,22^ The CECA is a comprehensive semi-structured interview whose standardized coding system attempts to improve the objectivity of ratings by basing them on the coder’s perspective (rather than relying on the participant’s judgment) and focusing on concrete descriptions rather than perceptions or emotional responses to the questions, together with considering the context in which the adverse experience occurred.

In our adapted coding scheme, the anchor points of the scale differ for each victimization category, with some focused more on the severity of physical injury that is likely to have been incurred during victimization exposure (crime victimization, family violence, maltreatment), while others are more focused on the frequency of occurrence of victimization (peer/sibling victimization and internet/mobile phone victimization), the physical intrusiveness of the event (sexual victimization), or the pervasiveness of the effects of victimization (neglect). This reflects the different ways in which severity has previously been defined for different types of victimization.^20,23^ (Given that our sample comprises twins, we also coded if any of the victimization events experienced by each twin had been perpetrated by their co-twin, as it is possible that growing up with a genetically related, same-age child could increase or decrease sibling victimization rates.) Each twin’s dossier was evaluated separately, and we did not use information provided in the co-twin’s dossier about their own or shared victimization experiences to rate direct or witnessed violence exposure for the target twin. High levels of inter-rater reliability were achieved for the severity ratings for all forms of victimization: crime victimization (intra-class correlation coefficient [ICC] = 0.89, p < 0.001), peer/sibling victimization (ICC = 0.91, p < 0.001), internet/mobile phone victimization (ICC = 0.90, p < 0.001), sexual victimization (ICC = 0.87, p < 0.001), family violence (ICC = 0.93, p < 0.001), maltreatment (ICC = 0.90, p < 0.001), and neglect (ICC = 0.74, p < 0.001).

The ratings for each type of victimization were then grouped into three classes: 0 – no exposure (score of 0), 1 – some exposure (score of 1, 2 or 3), and 2 – severe exposure (score of 4 or 5) due to small numbers for some of the rating points. Combining ratings of 4 and 5 is also consistent with previous studies using the CECA, which have collapsed comparable scale values to indicate presence of ‘severe’ abuse.^20,22,24,25^ The adolescent poly-victimization variable was derived by summing all victimization experiences that received a code of ‘4’ or ‘5’ (i.e., severe exposure): 64.6% of adolescents had zero severe victimization experiences; 19.2% had 1; 9.4% had 2; 4.5% had 3; 1.5% had 4; 0.5% had 5; and 0.2% had 6 severe victimization experiences. Due to small numbers in some of the groups, we collapsed this variable into ‘0’ not victimized, ‘1’ experienced 1 type of severe victimization, and ‘2’ poly-victimized (experienced 2 or more types of severe victimization).

**References**

1. Trouton A, Spinath FM, Plomin R. Twins Early Development Study (TEDS): a multivariate, longitudinal genetic investigation of language, cognition and behavior problems in childhood. *Twin Res*. 2002;5(5):444-448.

2. Moffitt TE, E-Risk Study Team. Teen-aged mothers in contemporary Britain. *J Child Psychol Psychiatry*. 2002;43(5):1-16.

3. CACI. *ACORN user guide*. London, UK: CACI Information Services; 2006.

4. Caspi A, Taylor A, Moffitt TE, Plomin R. Neighborhood deprivation affects children’s mental health: environmental risks identified in a genetic design. *Psychol Sci*. 2000;11(4):338-342.

5. Polanczyk G, Moffitt T, Arseneault L, et al. Etiological and clinical features of childhood

psychotic symptoms. *Arch Gen Psychiatry.* 2010;67(4):328-338.

6. Poulton R, Caspi A, Moffitt TE, et al. Children’s self-reported psychotic symptoms and adult schizophreniform disorder: a 15-year longitudinal study. *Arch Gen Psychiatry.* 2000;57:1053-1058.

7. Schreier A, Wolke D, Thomas K, et al. Prospective study of peer victimization in childhood and psychotic symptoms in a non-clinical population at age 12 years. *Arch Gen Psychiatry.* 2009;66:527-536.

8. Kelleher I, Connor D, Clarke MC, Devlin N, Harley M, Cannon M. Prevalence of psychotic

symptoms in childhood and adolescence: a systematic review and meta-analysis of

population-based studies*. Psychol Med.* 2012;42:1857-1863.

9. Zammit S, Kounali D, Cannon M, et al. Psychotic experiences and psychotic disorders at age 18 in relation to psychotic experiences at age 12 in a longitudinal population-based cohort study. *Am J Psychiatry.* 2013;170:742-750.

10. March JS, Parker JD, Sullivan K, et al. The Multidimensional Anxiety Scale for Children (MASC): factor structure, reliability, and validity. *J Am Acad Child Adolesc Psychiatry.* 1997;36(4):554-565.

11. Kovacs M. *Children’s Depression Inventory (CDI) manual*. Multi-Health Systems; 1992.

12. Kim-Cohen J, Moffitt TE, Caspi A, Taylor A. Genetic and environmental processes in young children’s resilience and vulnerability to socioeconomic deprivation. *Child Dev.* 2004;75(3):651-668.

13. Fisher HL, Caspi A, Moffitt TE, et al. Measuring adolescents’ exposure to victimization: The Environmental Risk (E-Risk) Longitudinal Twin Study. *Dev Psychopathol.* 2005;27(Special Issue 4pt2):1399–1416.

14. Finkelhor D, Hamby SL, Turner HA, Ormrod RK. *The Juvenile Victimization Questionnaire: 2nd Revision (JVQ-R2)*. Durham, NH: Crimes Against Children Research Center; 2011.

15. Hamby S, Finkelhor D, Ormrod D, Turner H. *The comprehensive JVQ administration and scoring manual*. Durham, NH: University of New Hampshire, Crimes Against Children Research Center; 2004.

16. Finkehor D, Hamby SL, Ormrod RK, Turner HA. The Juvenile Victimization Questionnaire: reliability, validity, and national norms. *Child Abuse Negl.* 2005;29:383-412.

17. Radford L, Corral S, Bradley C, Fisher H, Bassett C, Howat N, Collishaw S. *Child abuse and neglect in the UK today*. London: NSPCC; 2011.

18. Radford L, Corral S, Bradley C, Fisher HL. The prevalence and impact of child maltreatment and other types of victimization in the UK: findings from a population survey of caregivers, children and young people and young adults. *Child Abuse Negl.* 2013;37(10):801-813.

19. Caspi A, Moffitt TE, Thornton A, et al. The life history calendar: A research and clinical assessment method for collecting retrospective event-history data. *Int J Methods Psychiatr Res.* 1996;6:101-114.

20. Bifulco A, Brown GW, Harris TO. Childhood Experience of Care and Abuse (CECA): a retrospective interview measure. *J Child Psychol Psychiatry.* 1994;35(8):1419-1435.

21. Bifulco A, Brown GW, Neubauer A, Moran P, Harris T. *Childhood Experience of Care and Abuse (CECA) training manual*. London: Royal Holloway College, University of London; 1994.

22. Bifulco A, Brown GW, Lillie A, Jarvis J. Memories of childhood neglect and abuse: Corroboration in a series of sisters. *J Child Psychol Psychiatry.* 1997;38:365-374.

23. Barnett D, Manly JT, Cicchetti D. Defining child maltreatment: The interface between policy and research. In D Cicchetti & SL Toth (Eds.), *Child* *abuse, child development, and social policy* (pp. 7-74). Norwood, NJ: Ablex; 1993.

24. Bifulco A, Brown GW, Moran P, Ball C, Campbell C. Predicting depression in women: the role of past and present vulnerability. *Psychol Med.* 1998;28:39–50.

25. Fisher HL, Bunn A, Jacobs C, Moran P, Bifulco A. Concordance between mother and offspring retrospective reports of childhood adversity. *Child Abuse Negl.* 2011;35(2):117–122.
